# The Impact of Dementia Caregiving on the Health of the Spousal Caregiver

**DOI:** 10.3390/medicina62040796

**Published:** 2026-04-21

**Authors:** Donna de Levante Raphael, Lora J. Kasselman, Wendy Drewes, Isabella Wolff, Luke Betlow, Joshua De Leon, Allison B. Reiss

**Affiliations:** 1 Alzheimer’s Foundation of America, New York, NY 10001, USA; draphael@alzfdn.org (D.d.L.R.); wadrewes27@gmail.com (W.D.); izzywolff555@gmail.com (I.W.); lbetlow26@newarka.edu (L.B.); 2 Department of Medical Sciences, Hackensack Meridian School of Medicine, Nutley, NJ 07110, USA; 3 Department of Medicine, NYU Grossman Long Island School of Medicine, Mineola, NY 11501, USA; joshua.deleon@nyulangone.org; 4 Department of Foundations of Medicine, NYU Grossman Long Island School of Medicine, Mineola, NY 11501, USA

**Keywords:** dementia, caregiver outcomes, mental well-being, burden, psychological intervention, social stigma

## Abstract

Dementia caregiving represents a major public health challenge, with spousal caregivers assuming the greatest burden. Spouses, themselves typically older adults, provide high intensity, long-term, and largely unpaid care across all stages of cognitive decline. Despite their central role in dementia care, the health consequences experienced by spousal caregivers remain insufficiently characterized in the literature and inadequately addressed in clinical and public health practice. This structured narrative review synthesizes current evidence on the multidimensional impact of dementia caregiving on the physical, psychological, cognitive, social, and financial health of spousal caregivers. It further contextualizes these consequences within the trajectory of dementia progression, and identifies interventions, support systems, and policy considerations necessary to mitigate caregiver burden. Spousal caregivers experience disproportionate burden due to continuous, escalating responsibilities that often mirror the progressive deterioration of their partners. Emotional burdens, including uncertainty during pre-diagnostic stages, role strain, conflict, loss of intimacy, and anticipatory grief. Physically, spouses endure musculoskeletal strain, sleep disruption, poor nutrition, and heightened frailty risk. Psychologically, spousal caregivers exhibit elevated rates of depression, anxiety, loneliness, and stress-related disorders. Socially, caregivers experience substantial isolation, stigma, and erosion of social networks. Financial hardship, including early retirement, reduced employment, and uncompensated care hours, further exacerbate stress. Evidence suggests that chronic caregiving stress contributes to biological changes such as immune dysregulation, inflammation, acceleration, aging, and potential cognitive decline in caregivers themselves. Caregiver burden influences patient outcomes as evidenced by increased emergency department use, falls, and earlier institutionalization in persons with dementia whose caregiver is subjected to a high burden. Current care models rarely include routine, caregiver assessment or structured guidance following diagnosis, resulting in substantial unmet needs. Effective mitigation requires integrated, stage-sensitive interventions, including psychosocial support, caregiver education, respite services, culturally tailored programs, and digital health tools, alongside broader policy reforms to reduce financial and structural barriers.

## 1. Introduction

Alzheimer’s disease and related dementia are progressive and debilitating syndromes primarily affecting older adults. The consequences of these conditions fall upon not only those diagnosed, but also to the individuals around them [1]. Caregivers that hold a particularly intimate relationship with the patient face more intense repercussions to the burdens of dementia [2]. As dementia prevalence continues to climb nationally, with over 7 million individuals in the United States currently living with the condition, concern grows for the unintended health consequences to spouses and partners [2,3]. The burden of caregiving falls disproportionately on spouses, who often provide daily, unpaid care over many years. These caregivers become the frontline in managing the disease, supporting their partners through emotional, behavioral, and physical decline. While caregiving can often offer a sense of purpose and deepen marital bonds, it is also associated with extensive psychological, physical, social, and economic burdens. Spousal caregivers often face chronic stress, health deterioration, and increased risk of mortality. This review synthesizes the current evidence on the impact of dementia caregiving on spousal caregivers’ health outcomes and explores the basic trajectory of dementia caregiving roles across disease stages, and spectrum of burdens faced. This literature review further evaluates the resultant physical, mental, and psychosocial health consequences and identifies interventions and public health strategies aimed at mitigating caregiver harm.

## 2. Methodology

This study utilized a comprehensive multidisciplinary narrative literature review approach. A narrative approach was selected rather than a systemic one due to the dearth of data-driven research on our specific sub-population, demanding a robust paper which addresses our current understanding of specifically the spousal caregiving population. Peer-reviewed literature published between 2012 and 2025 was identified through comprehensive searches of PubMed, Scopus, PsycINFO, Cumulative Index to Nursing and Allied Health Literature (CINAHL), and Web of Science to identify peer-reviewed observational, longitudinal, and statistical research papers. Search terms used in various combinations included “spousal caregiver,” “dementia caregiving,” “Alzheimer’s disease,” “caregiver burden,” “psychological stress,” mental health,” “cardiovascular risk,” “sleep disturbance,” and “cognitive decline.”

Studies were selected based on five criteria: focus on dementia, publication within the specified year range, inclusion of spouse in the sample population, and discussion on an emotional, physical, social, or financial dimension of burden. Reviews, pilot studies, and other exploratory reports were included when they were directly relevant to the research question and provided substantive information on caregiver burden, as these sources can offer useful contextual and early-stage evidence in a developing literature. Papers that were outdated relative to our current knowledge of dementia/caregivers, contained no conclusive evidence, or did not have a representative population were excluded. After compiling our final set of papers, findings were organized under our four perspectives of physical, social, emotional, and biological. Common patterns and recurring themes were then identified within each domain, which guided the development of the subsections in the review.

## 3. Dementia Progression and the Evolving Role of Spousal Caregivers

### 3.1. Overview of Dementia

The expanding elderly population coupled with the increasing prevalence of dementia make understanding the progression of this disease and its impact on caregivers, families and society a pertinent public health issue [1,2]. Dementia affects approximately 11% of U.S. adults age 65 years and above [3]. Underdiagnosis of dementia indicates that this number may be a low estimate [4].

Dementia worsens over time at varying rates [5]. There are generally three main phases of dementia: mild/early, moderate/middle, and severe/late. Clinicians have further subdivided this categorization into 7 stages to better represent the continuum and gradual development of cognitive and behavioral symptoms [6]. Each stage may manifest differently for each patient, depending upon subtype. Dementia is an umbrella term that encompasses multiple possible etiologies that manifest as cognitive decline [7]. While it is difficult to define specific, concrete characteristics of each phase of dementia, key characteristics of the main subtypes and an outline of the general course provide a foundation for later discussion on the caregiving experience. This framework can be applied to anticipate the primary spousal caregiver responsibilities accompanying each phase. This will allow us to contextualize a holistic understanding of the health outcomes for spousal caregivers as the experience is turbulent and progressive in nature, paralleling the arc of symptoms for care-recipients.

In general, dementia is defined as a progressive deterioration of memory, judgment and cognition that occurs due to damage to the nerve cells of the brain [8,9]. Accumulating damage ultimately impacts the ability to perform daily tasks, navigate the environment and live independently. Impaired ability to complete everyday necessities is a key component to the disease and the role filled by caregivers [10]. There are many types of dementia, usually defined by the specific cause of brain damage, as well as diseases that result in dementia-like symptoms. To add to the continuum, a common type of dementia is mixed dementia where individuals may have multiple forms of dementia [11,12].

### 3.2. Diagnosis

The diagnosis of dementia is made after extensive clinical evaluation generally involving full physical examination, blood testing and neuropsychological assessment [13,14]. The cause of dementia is only established definitively with histopathological evaluation at autopsy, but in recent years, biomarkers have become increasingly accurate tools for the diagnosis of neurological disorders [15,16]. Specifically, biomarkers in the cerebrospinal fluid of patients with AD have inspired similar work for disorders such as Parkinson’s disease, frontotemporal dementia, and Lewy bodies dementia [17].

For medical assessments and evaluation, several cognitive tests may be used to measure different domains of cognition [18,19]. A key factor in diagnosing dementia is family or friends noting certain behavioral changes in the patient [20]. There is often a lag in the time from spousal realization that dementia symptoms are being exhibited and physician consultation [21]. There is a delineation between mild cognitive impairment (MCI) and dementia. In MCI, cognition declines but ability to perform activities of daily living remains intact [22]. While MCI does not always lead inevitably to dementia, it is estimated that between 10 and 20% of persons with MCI over the age of 65 years will develop dementia within a one-year timeframe [23,24]. It is important for spousal caregivers, the ones who tend to spend the most time with their partner, to recognize changes indicating worsening in order to obtain resources and information to prepare for increasing burden [25].

Spousal caregivers are able to observe subtle cognitive changes within their care-recipient, often prompting the initial medical appointment that leads to diagnosis [26]. On average, time from appearance of symptoms to formal diagnosis takes 3 years for late-onset dementia and over 4 years for early-onset dementia [27]. In many cases, spouses begin assuming caregiving responsibilities prior to formal diagnosis [28]. During this pre-diagnostic period, caregivers navigate uncertainty, including evolving symptomatology, limited prognostic information, and a lack of structured guidance on managing emerging care needs [29,30]. These early experiences are compounded by the progressive nature of dementia and underscore the central role of spousal caregivers within the continuum of dementia care. The specific impacts of caregiving on the health outcomes of spouses remain under-characterized in the literature, despite this population being a large proportion of family caregivers of people with dementia [31,32]. This review aims to synthesize the current body of literature regarding spousal caregiving burdens, the known health outcomes, and what is lacking in the field.

### 3.3. Classification of Caregivers

The primary caregiver for persons with dementia is most often either an adult child or spouse/partner and 30% of caregivers are age 65 years or older [3,33,34,35]. The landscape of dementia caregiving is shaped not only by the stage and rate of the patient’s cognitive decline, but also by the relationship between the caregiver and the patient [36,37,38]. Understanding the evolution of the caregiver role across progressive stages of dementia is crucial for contextualizing the psychosocial and health impacts that follow, especially in the case of spousal caregiving for AD and related dementias.

Primary caregivers assume the major responsibilities associated with daily caregiving [39,40,41,42]. Spousal caregivers in particular tend to provide sustained and intimate support, often alone and with limited formal assistance [37,43,44]. Secondary caregivers such as siblings, friends, and extended family may contribute periodic or supplementary caregiving [45,46]. Professional caregivers (paid aides, visiting nurses, etc.) are frequently involved as cognitive decline and related symptoms worsen in order to supplement spousal caregiving [47,48]. These professionals not only administer in-home treatment and care but also act as resources in educating and training spousal and other family caregivers [49,50]. Hybrid models, blending informal and formal care, are common, but vary widely based on individualized needs, socioeconomic background, and access to healthcare resources [51,52,53].

### 3.4. Stages of Dementia Caregiving and Role Evolution

In the early (mild) phase of dementia, caregiving is often focused on subtle assistance such as providing reminders, daily task supplementation, and monitoring behavior in order to optimize functioning [54,55,56]. Caregivers often struggle to balance oversight with respect for autonomy, especially in cultures or family contexts where denial or non-disclosure is prevalent [57,58,59,60]. During this period, the caregiver frequently transitions into a surrogate decision maker, often without formal preparation or training to prepare them for these developing responsibilities [61,62]. At this stage, most caregivers retain leisure or free time in which accessing and internalizing information resources to inform the caregiving process are vital in preparation for oncoming developments of disease progression [63,64].

As cognitive decline progresses to the middle (moderate) stage, caregivers often assume an increasingly central role in managing the daily life of the patient. Supervision intensifies to cover personal care and administration of treatments and medication [39,65,66]. Behavioral and emotional symptoms such as agitation, aggression, apathy and depression can emerge, often together, and require mitigation by the caregiver [67,68,69,70]. The emotional dynamic also shifts, with spousal intimacy complicated by emerging dependency [71,72,73,74,75].

Caregivers at this middle stage may find themselves with a growing need to reach out and engage actively with healthcare and community resources [76,77,78]. However, fragmented and complicated means of access can make it difficult to find information and assistance, leaving needs unmet [79]. Further, a lack of instruction in how to communicate with the dementia patient may add to caregiver stress and feelings of uncertainty [80]. Despite obstacles, caregivers may continue to find ways to share enjoyable activities with the patient [81].

In the late (severe) stage of Alzheimer’s and related dementia, caregiving becomes round-the-clock, encompassing all activities of daily living (dressing, bathing, transportation, etc.), often including palliative care [82,83,84]. Caregiving becomes more physically and emotionally demanding [85].

At this stage of profound decline, spousal caregivers often face emotional and psychological strain as the relationship dyad deteriorates into one-sided caregiving in which the patient retains little to no self-sufficiency or autonomy [86,87]. This development is commonly marked in caregivers by anticipatory grief, loneliness, and the erosion of mutual interaction [88,89,90,91].

Decision-making at this stage typically extends to complex medical and end-of-life dealings, a responsibility many caregivers feel ill-equipped to manage without adequate support systems or intervention strategies [92,93,94,95]. In many cases, spousal caregivers step out of their central caregiving role during end-of-life care, relinquishing responsibilities to healthcare professionals and secondary caregivers [96,97,98,99]. Importantly, this change may not be an option for caregivers from lower-income backgrounds who do not have the financial resources to bring in professional care [100,101,102].

**Figure 1:**
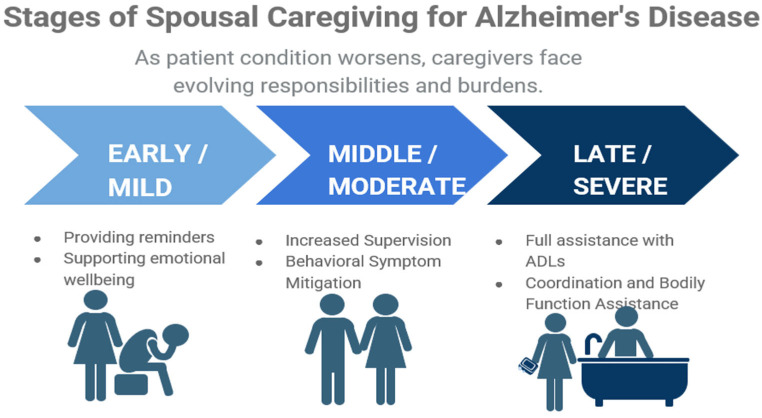
Depiction of the progressive nature of caregiving responsibilities experienced by spouses of individuals living with Alzheimer’s disease. As dementia advances, caregivers take on increasingly complex and demanding tasks. The diagram uses three indicators: Early/Mild, Middle/Moderate, and Late/Severe to depict the evolving caregiving role across the clinical trajectory of Alzheimer’s disease and related dementia.

In summary, dementia caregiving tends to unfold in sequential stages that mirror the decline of the person needing care (Figure 1).

### 3.5. Caregiver Role Adaptation over Time

Throughout the dementia trajectory, caregiver responsibilities shift in parallel with the progression of cognitive decline of the patient [103]. Decision-making authority gradually shifts from shared to unilateral, while emotional dynamics move from partnership to protective management [104,105]. Transitions such as hospitalization, placement into long-term care, or the patient’s death force further psychoemotional changes, often accompanied by unresolved guilt or ambivalence [106,107,108]. Recognition of these adaptive pressures is vital for developing responsive interventions in order to support caregivers in all stages of disease progression.

## 4. Multidimensional Burden of Spousal Caregiving

### 4.1. Domains of Burden

Spousal caregivers often provide care over multiple years of a downward trajectory, often spanning 5 years or more [44,109,110,111]. Primary spousal caregivers experience a major shift in roles to their care-recipient, from a reciprocal partner to a singular provider [37,112,113]. The burdens of this subgroup are multifaceted and require focused research to better understand the implications of these singular additional factors in caregiving. The aim of this section is to identify the emotional, physical, social, and financial burdens faced by spousal caregivers to help inform targeted interventions and policies to best protect this population from worsening health outcomes [114].

### 4.2. Emotional Burdens

#### 4.2.1. Uncertainty from Diagnosis

The early stages of subtle cognitive decline can mark the beginning of increased demand and stress on the caregiver [115]. The pre-diagnosis period, which can span several years, is marked by uncertainty and growing concern about the health of the spouse [29,116]. Since functional ability is not impaired at this early stage, it is not well-recognized and there is little formal support for the caregiver [117,118,119]. The close proximity of the spouse and partner places the spouse in a care-giving role by default [120,121,122]. The subtle and incremental increase in caregiver responsibilities continues for varying periods until a threshold is reached and the partner urges their loved one to seek medical care and evaluation [3,123,124,125,126]. Prolonged ambiguity combined with increasing responsibilities culminating in a formal diagnosis can be met with a range of reactions: shock, denial, uncertainty, and more [127,128,129]. The pre-diagnostic phase of spousal caregiving is sparsely defined and understood in the larger caregiving experience, particularly in health outcomes. After diagnosis, family members report a lack of structure and formal resources [130]. Specifically, many couples reference the meager information provided about the disease course and their lack of knowledge of what is to come [131].

#### 4.2.2. Role Conflict and Role Strain

Caregiver activities of spousal caregivers bring both role strain and role conflict [132]. Role strain is when the act of managing all the responsibilities of a caregiver exceeds the caregiver’s capacity, causing emotional distress. As time progresses, care only intensifies, and without instilling proper mitigation strategies and coping mechanisms, the strain will intensify [133]. Specifically, neuropsychiatric behavioral symptoms are cited to increase strain of dementia caregivers [134]. Role conflict is when the role of caregiver begins to conflict with the other roles a spouse may hold [135]. This concept usually emerges as strain increases because at that point spouses need to dedicate more time to their care-recipient, eventually conflicting with their other roles. This conflict is particularly prevalent among retired spousal caregivers who can take on hours equivalent to full-time employment due to available time and co-residency [37,136]. The role of a spousal caregiver increases the burden of role conflict and role strain, and this is attributed to close proximity in living arrangements and intertwined social networks [121,137,138]. Despite its clinical relevance, this complexity and its health outcomes have not been investigated in the spousal caregiver population. Spousal role conflict and strain merit greater attention as these may be a potent cause for poor health outcomes in spousal caregivers and is a possible target for mitigation strategies to improve overall wellbeing [139].

#### 4.2.3. Loss of Emotional Connection with Spouse

Being a caregiver for a spouse with dementia changes the relationship, further complicating the ability to deliver care [140]. As one partner devotes more time and energy to the other due to caregiving tasks, a decline in relationship quality occurs, straining the caregiver from a lack in emotional satisfaction [141,142]. This emotional burden is closely linked to the care-recipient’s behavioral symptoms such as apathy and agitation, which impact a caregiver’s well-being [143,144,145,146,147]. The relationship turning from reciprocal partnership to unidirectional caregiving results in a lack of intimacy and a decrease in the sense of responsibility for each other. These emotional repercussions are important to note because they cause caregiving to be a more strenuous and emotionally taxing endeavor [71,148]. A modulator in the toll of caregiving is the quality of marital bonds prior to the emergence of dementia [149]. Loneliness is common in spousal caregivers, even early in the trajectory of cognitive decline [150]. Kotwal et al. found that for those couples who share a solid emotional connection, the relationship between spousal cognitive decline and increased loneliness is strong, while this association is weaker for couples who were not emotionally close prior to cognitive changes [151]. Due to the time invested in caregiving as well as the changing relationship with their spouse, it is common for spouses to report a loss of identity and that their life lacks meaning [37,43]. Using this defined relationship for partners may help to guide interventions and formulate mitigation strategies to boost the positive aspects of caregiving [152,153].

#### 4.2.4. Progressions Towards Anticipatory Grief

Anticipatory or pre-death grief is the sentiment of worry regarding losing your loved one. There is a relationship between anticipatory grief and worsening health outcomes for spousal caregivers [154,155]. Spousal caregivers are particularly prone to experiencing anticipatory grief, exhibiting more intense sentiments of anticipatory grief than other types of caregivers [156,157]. The outcome results in more burden, greater use of acute healthcare resources and increased depressive symptoms and anxiety [155,158].

### 4.3. Physical and Somatic Burdens

#### 4.3.1. Performing Physically Challenging Tasks

An important consideration is that on average spousal caregivers are about 72 years of age while the larger dementia caregiving population is on average 53 years old. This means that the spousal caregiver population will be less robust in handling the physical burden and may have their own frailty issues [159,160,161]. In earlier stages of dementia, the physical strain on the caregiver may be somewhat limited to taking on household duties like cleaning the dishes or taking out the trash. After some time, the spousal caregiver may need to perform more demanding tasks, such as clothing and bathing that require bending, lifting and maneuvering. Finally, the most intense physical burden may involve aiding in mobility and supporting body weight [162,163]. Musculoskeletal disorders are the largest cause of care burden due to the physical strain caregiving requires [164,165]. However, specific relationships with spousal caregiving and physical pain have not been investigated nor defined [166]. This topic needs attention as spousal caregivers tend to be of an older age and to be living with the care-recipient, making them at higher risk for health outcomes.

#### 4.3.2. Sleep Disruption

Caregivers of persons with dementia are particularly vulnerable to sleep deprivation and sleep disturbances [167,168]. A meta-analysis from Gao et al. revealed that on average, caregivers of persons with dementia lose approximately 2–4 h of sleep each week [169]. A meta-analysis from Cho et al. found that high-intensity spousal caregivers are more likely to experience sleep disturbances than low-intensity caregivers and a cross-sectional study from Koyanagi et al. encompassing 58 countries had similar findings [170,171].

The experience of sleep is unique for the spousal caregiver but there is a dearth of data on this aspect of the intimate relationship. An example of this knowledge gap is the lack of study of the ways in which sharing a bed or following different sleep habits, like moving to a separate bedroom, can augment the physical sleep burden [172]. In a scoping review investigating sleep and caregiver burden of patients with dementia, out of the 24 sources in the study, only 25% of them examined a population specifically of spousal caregivers [173].

### 4.4. Social Burdens

The most obvious social burden of spousal caregiving is a decline in social networks, particularly during high-intensity phases of caregiving [154,174]. The extent of social network loss due to caregiving is dependent upon disease course and severity [175,176,177,178]. Caregiving is very time consuming, leaving less hours for socializing [179,180].

Social isolation is not only a result of the large chunks of time taken up by caregiving but can also stem from trepidation in relation to public situations with the care-recipient [37,181]. This can limit participation in family outings and cause caregivers to fear losing their personal identity. The unpredictable behavior of the person with dementia and inability to control the person or keep the person safe from harm are factors that may discourage attempts to socialize [182,183]. The ways in which the caregiver experiences social impacts vary based on cultural and gender background and this variation can inform choice of intervention [184,185,186,187]. Social isolation may be a compound result of caregiving demands, cultural background, and regional differences in family dynamics [188].

Family stigma, defined as stigma extended to the family members of stigmatized individuals, is a social burden that has been shown to reduce quality of life [189,190,191]. A lack of education and public awareness leads to these ramifications, leaving caregivers at risk to face misunderstanding and lack of support managing all their responsibilities [100].

Spousal caregivers may achieve a better balance with greater social engagement if they have multiple support systems in place, including peer support [177,192]. Online platforms for socializing may also be helpful and are convenient and accessible without travel [193,194]. Group activities may help caregivers and dementia care recipients to relieve loneliness [195,196,197].

### 4.5. Financial Burdens

Spousal caregivers often face significant financial burdens due to the time and resources required for caregiving [198,199]. While estimates vary, the Family Caregiver Alliance estimates that family caregivers in general spend an average of 24.4 h per week on caregiving tasks while the spousal caregiving population spends on average of 44.6 h a week on care [200]. Persons with dementia need an average of 27 more hours of caregiver time per month than persons without dementia who require care [3,201]. As a result of devoting these hours to caregiving, the caregiver may retire early, or reduce or leave paid employment, leading to financial strain [202,203]. In addition, caregivers need to keep records of medical expenses, budget for new medical expenses and navigate healthcare resources, which all take up more time. These responsibilities contribute to broader economic impacts, as caregivers experience poorer health, limited social engagement, and reduced productivity [204]. Lack of governmental support and other systems upon which to rely for budget management can worsen financial strain [100,205,206]. Studies on time constraints as a spousal caregiver and the effects on their financial situation would better characterize this major stressor and inform concrete ways to support spousal caregivers.

## 5. Health Consequences for Spousal Caregivers

### 5.1. Spousal Caregiver Health Outcomes

**Figure 2:**
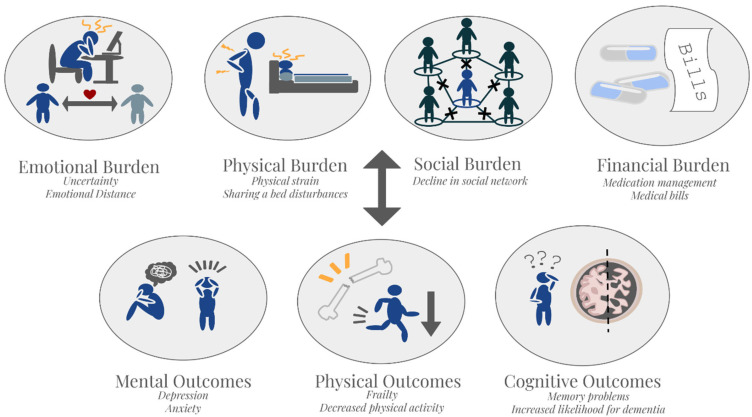
Caregiver burdens and consequences to the carer. Illustrates the interconnected burdens and potential resulting detrimental health effects experienced by spousal caregivers of individuals living with dementia. Each oval depicts a core domain of strain or an associated health outcome, emphasizing how these burdens interact and compound over time.

Spousal caregivers of individuals with dementia face significant health consequences across physical, psychological, and social domains (Figure 2). Prolonged caregiver stress is associated with increased risk of chronic conditions such as cardiovascular disease, sleep disturbances, immune suppression, and accelerated biological aging [207,208,209]. According to data compiled by the Family Caregiver Alliance, 27% of spousal caregivers rate their overall health as fair or poor [210]. Socially, caregivers often face isolation, reduced social participation, and relationship strain, particularly as the spousal dynamic shifts from reciprocal partnership to unidirectional caregiving [211,212]. These outcomes collectively lead to reduced quality of life and increased healthcare utilization [213]. Given that many spousal caregivers are older adults themselves, these burdens pose risks not only to caregiver health, but also to the sustainability of home-based dementia care.

### 5.2. Psychological and Mental Health Outcomes

Spousal caregivers are at higher risk for poor mental health [214]. Specifically, Torres et al. analyzed data on spousal caregivers of patients with dementia from 2000 to 2018 extracted from the Health and Retirement Study and found that for those living with a spouse who had cognitive impairment or dementia, 12% of the women noted depressive symptoms and 15% experienced loneliness while for the men, 7.3% noted depressive symptoms and 10.3% experienced loneliness [215]. In a separate analysis from Kotwal et al., researchers also used the Health and Retirement Study and evaluated 3666 married couples enrolled between 2014 and 2016 [151]. They found that nearly one in three spouses of patients with dementia experienced loneliness and one in six experienced depression. Using longitudinal data from the Health and Retirement Study, Liu et al. showed that depressive symptoms occurred even in spouses who had recently taken on caregiver responsibilities [216]. The development of depressive symptoms is closely aligned to the perceived health of the care-recipient [217,218]. Spousal caregivers have higher levels of loneliness and social isolation than both non-spousal carers and other informal carers [90].

Sources of anxiety for a spousal caregiver range from a lack of information regarding progression of disease, to the need to make important medical decisions, and to worries regarding the future [219,220]. Additional support, both formal and informal and particularly from the healthcare system can help spouses navigate the myriad challenges and obstacles [175,221,222].

Mental health struggles of spousal caregivers have major repercussions to the overall healthcare system [223,224]. Caregivers with mental health problems may be more likely to seek professional care for their care recipient. Caregiver depression is highly associated with emergency department utilization for the dementia patient [93,225,226,227]. A cross-sectional study from Sullivan et al. using Medicare data found that hospice use was higher for dementia patients whose caregiver was having emotional difficulties [228]. Legget et al. found that caregivers were more likely to have emotional difficulties if their care recipient with dementia had experienced a fall in the prior month [229]. Additionally, caregiving for a partner with dementia can increase the probability that the caregiver will have an overnight hospitalization compared to a non-caregiving spouse [230]. For these reasons, there is an urgent public health matter to address the mental health needs of caregivers in order to support the overall population and to not overwhelm the greater healthcare system [231].

### 5.3. Physical Health Outcomes

Caregiving burden often results in a decline in the caregiver’s level of physical activity. Under the stress of high intensity caregiving, caregivers engage in less physical activity compared to those under the lesser demands of low-intensity caregiving [154,170,232]. A separate study found that leisure-time physical activity was lower in spousal caregivers versus non-spousal caregivers [233]. These findings are a call to action as physical activity can help reduce caregiver burden [234,235,236,237,238]. Egan et al. have sought a low-cost and practical solution using technology to promote physical activity via a digital health app specifically for caregivers of persons with dementia [239]. Research still requires a specific focus on the spousal caregiving population in order to support helpful interventions and health guidelines that providers can recommend [240].

Poor sleep is a tangible physical health issue for caregivers that contributes to burden. Specifically, poor sleep can result from poor mental health, low physical activity, and caregiver load [241,242,243]. A moderator for physical activity and sleep is caregiving intensity [140,244]. A study from Chu and Jang found depression to be a key mediator of poor sleep quality in spousal caregivers of persons with dementia [245]. Sleep programs involving both the patient and caregiver may offer relief from sleep difficulties [246].

In addition to poor sleep, eating habits of spousal caregivers may be impacted with poor dietary choices and patterns of emotional eating [247,248].

Frailty, defined as increased vulnerability to illness and injury, coupled with the decreased ability to recover from such ailments is a major issue in spousal caregivers particularly due to their older age [249,250]. Research has confirmed significant increases in spousal caregivers’ frailty index over time compared to the non-caregiver population. The extent of frailty is greater in addition to the speed of progression for spousal caregivers [161,251,252]. Factors leading to frailty are attributed to multiple circumstances. Decline in physical activity is a negative health outcome of caregiving and also a related factor for the incidence of frailty [253]. High-intensity spousal caregivers have reported performing less physical activity compared to their low-intensity counterpart [170]. Another important factor for frailty is caregiver burden. Wennberg et al. linked more enjoyable caregiving to lower risk of frailty in the older caregiver [254].

The older age range of the spousal caregiver population inherently brings a greater likelihood that caregivers will be managing their own chronic conditions in addition to their caregiving responsibilities [255,256]. Time invested in providing care to their partner may result in loss of resources or less time management for their own ailments. As a result, research has shown adverse effects on the relationship between spousal caregivers that have chronic conditions and their own personal health outcomes [162,257]. These findings support the idea that caregiving poses a particular risk to the spousal population.

### 5.4. Cognitive Outcomes of Caregivers

Some studies show that cognitive decline is prevalent in spousal caregivers while others have not found this association [258,259]. Brown et al. showed that caregiver burden negatively affects self-reported subjective cognitive decline while a systematic review from Turner et al. also found higher likelihood of subjective cognitive decline in spouses of persons with dementia [260,261]. Wu-Chung et al. showed that spousal caregivers have worse cognitive outcomes and increased risk for developing dementia [262]. They propose that a biopsychosocial model is a highly appropriate way to assess and analyze the cognitive risks associated with being a spousal caregiver. García-Castro found that informal caregivers had a higher level of memory and executive function [263]. Researchers have not yet resolved this issue.

## 6. Intervention and Mitigation

Supporting dementia caregivers necessitates interventions that are flexible, individualized, and sensitive to the evolving demands of the caregiving role throughout each stage of caregiving [264]. Successful strategies span psychosocial, educational, structural, and technological forms [265,266]. Nonetheless, barriers to access and utilization of each type of resource persist, demanding approaches involving multiple forms.

### 6.1. Psychosocial Interventions

Psychosocial support such as structured support groups and mindfulness-based interventions demonstrate benefit in reducing stress, promoting resilience, and assuaging feelings of loneliness or isolation [267,268]. Models like the “Alzheimer’s Café” offer a social gathering with dual benefits of facilitating social interaction while also normalizing the caregiver experience [269,270]. However, stigma surrounding mental health, reluctance to prioritize personal needs, and cultural norms discouraging help-seeking behavior continue to limit participation, particularly among populations of older spousal caregivers [271,272]. Furthermore, mentalizing imagery therapy, consisting of psychotherapeutic exercises and mindfulness training, can positively impact the psychoemotional states of family dementia caregivers, reducing stress and improving resilience [273,274,275]. A systematic review from Yang et al. found that informal caregivers of patients with dementia receiving psychological therapy obtained psychological and emotional benefit from a variety of approaches including the use of breathing techniques, mindfulness training, self-care practices, and engaging with social support groups [276]. A cross-sectional study from Spain profiling family caregivers of people with dementia, found that spousal caregivers had a lower educational level and dedicated more hours per day to caregiving and would not place their partner in a care home while offspring caregivers had received more education, spent less hours per day administering care and were more likely to contemplate putting their parent into a care home [277]. This study highlighted caregiver profiling with incorporation of caregiver socio-demographics and individualized characterization of burdens of care in order to formulate tailored support and therapy.

### 6.2. Educational and Skill Building Programs

Educational interventions that equip caregivers with knowledge about dementia progression and techniques for behavior management have been shown to alleviate burden and improve care quality [278,279]. Resources such as the Alzheimer’s Foundation of America 24/7 Helpline, local caregiver training programs, and online modules (through AARP, Family Caregiver Alliance, etc.) offer practical guidance, emotional support, and information navigation [280,281]. These systems are especially critical for caregivers who feel isolated or overwhelmed throughout the difficult caregiving process [278,282,283,284,285]. However, disparities in literacy, education, and technological proficiency often constrain program efficacy. For many caregivers, especially those impacted by socioeconomic disadvantages, accessing and fully utilizing such programs remains a challenge [286,287].

### 6.3. Formal Support and Respite Services

Respite services, whether in-home, adult day programs, or institutional short-term care, provide crucial relief for caregivers [288,289,290]. Unfortunately, financial barriers, inconsistent local availability, and emotional resistance to delegating care (which is especially prevalent in spousal care dyads) impede uptake [51,286,291]. Immigrant and minority caregivers face additional hurdles, including language barriers and limited culturally appropriate services [286,292,293]. An interview-based study focusing on a cohort of Punjabi Sikhs found that tailored delivery and logistics of psychosocial interventions as well as cultural nuances to intervention content is necessary to more successfully produce positive outcomes amongst cultural groups with distinct views on gender, familial relationships, and attitudes towards gender identity and roles [294].

### 6.4. Technological Interventions

Digital tools including mobile applications, telehealth counseling, and online resources, present promising avenues for scalable caregiver support [295,296,297]. Tailored platforms like Olera.care show potential for enhancing user experience and personalizing resources—a major pitfall of many earlier technological interventions [298]. Older caregivers often encounter usability challenges, and existing tools frequently lack cultural and linguistic customization [287,299,300]. New developments include personalized immersive virtual environment technology (derived from virtual reality technology) which provides dementia-caregiver dyads with sensory experiences and interventions for psychosocial health. While preliminary in development and implementation, this technology shows promise in personalizing technological interventions for dementia caregivers [301,302].

### 6.5. Policy and Structural Support

Systemic supports such as caregiver tax credits, paid family leave, and expanded home care benefits present important and necessary supports in sustaining spousal caregivers throughout such a demanding time both emotionally and financially [303,304]. Still, navigating these systems is notoriously complex, with many caregivers unaware of their entitlements or deterred by detailed logistical processes encountered in order to utilize these benefits [51,79]. Current policy reform initiatives are well-meaning but may not adequately consider the specific needs of caregivers [79,305,306]. Beach et al. propose that reforms be instituted to broaden focus from merely expanding benefits to encompass simplifying access and providing clear, proactive guidance to caregivers [307]. Collaboration among groups supporting dementia caregivers and public health officials is vital to the implementation and success of public health strategies which could improve health equity and support for dementia caregivers [308].

Table 1 summarizes the many aspects of dementia caregiving by a spouse over time, highlighting dominant burden domains and health consequences for the caregiver and mitigating actions to ease the stress on the caregiving partner across stages of disease progression.

## 7. Future Considerations and Implications

### 7.1. Improving Responsiveness for Caregiver Health

Intense caregiving can bring adverse consequences for the caregiver, impacting mental, financial and physical well-being (Figure 1) [207,309,310,311,312,313]. Spousal caregivers reporting high-intensity caregiving exhibit significantly greater sleep disturbance, fatigue, and reduced physical activity compared to their low-intensity counterparts, signaling a need for routine screening and support strategies [170,314,315]. Furthermore, caregiver depression has been shown to increase the likelihood of emergency department use by dementia patients thus positioning caregiver well-being as an indirect but relevant determinant of patient outcomes [225,316].

Unfortunately, most dementia care models lack structured or routine caregiver assessments to indicate worsened burden in order to prompt intervention [317]. Clinical protocols to explicitly address the evolving state of caregiver health alongside the progression of the patient’s status can provide vital information to aid in designing mitigating strategies to preserve caregiver health [36,318]. Clinical adoption of tools like the Zarit Burden Interview or Caregiver Strain Index for routine caregiver assessments could enable earlier detection of worsened caregiver condition [319,320]. Additionally, many caregivers report that following a dementia diagnosis, they receive insufficient guidance regarding disease progression, decision-making, or resources [131,321]. The combined lack of caregiver check-ins and insufficient support signals a need for systematic interventions to ease the caregiving journey while monitoring and adjusting for changes that occur as the burden increases with progression of dementia.

### 7.2. Research Priorities

As biomarkers for early Alzheimer’s disease detection become increasingly accurate and prevalent, a parallel development in biological signature detection may be possible for monitoring and quantifying caregiver burden [322,323,324]. Specifically, spousal caregivers with elevated proinflammatory cytokine production demonstrate significantly higher levels of depressive symptoms and anticipatory grief [325,326]. These findings highlight the utility of integrating biomarkers into caregiver risk assessment and implementing these tools for quantifying the need for interventions supporting spousal caregivers, not just for patients.

The impact of caregiver burden and caregiver distress on care-recipient outcomes also demands further attention. Depressive symptoms in caregivers, coinciding with worsened mental health and even cognitive decline in these caregivers, have been associated with increased fall risk among care recipients [327,328]. Additionally, emotional burden has been linked to accelerated decisions around long-term care placement and potentially premature institutionalization [131,329,330]. These issues and difficulties highlight the reciprocal effects of caregiver burden on quality of care and patient/caregiver health. As a result, future research may be directed toward evaluating disease progression in the care recipient and dyadic interventions that jointly target patient and caregiver outcomes cohesively rather than independently so as to safeguard against these reciprocal issues.

## 8. Limitations

This narrative review has several limitations that should be considered when interpreting its findings. Since narrative reviews do not follow a fully systematic protocol, they typically lack a formal search strategy, standardized study selection process, and risk-of-bias assessment, which may increase the likelihood of selection bias and reduce reproducibility. The literature included is highly heterogeneous, spanning qualitative, cross-sectional, and longitudinal studies with varied populations, measures, and outcomes, limiting direct comparison and precluding quantitative synthesis. Additionally, much of the existing evidence is observational and may be influenced by confounding factors such as socioeconomic status and access to support. The review is also constrained by gaps in the literature, including the underrepresentation of diverse cultural groups and caregiving contexts. These limitations highlight the need for further research.

## 9. Conclusions

Many crucial aspects of life are impacted by spousal caregiving amid the lack of boundaries that ensues with dementia. As a result, emotional and physical hardships are common for the carer but may be somewhat alleviated with strong support. Mitigation strategies to avoid deterioration of spousal caregiver health have the potential to decrease the load on the healthcare system [331]. However, the current literature is not sufficient to determine how best to aid this population. The majority of studies state that spousal caregivers, in general, are at the greatest risk for health complications, and dementia caregivers are the ones who experience a disproportionate burden from caregiving [332,333]. It is hoped that this paper will highlight this knowledge gap and prompt research into best practices for positive health outcomes of spousal caregivers of those living with dementia, especially as this dyad continues to grow in size and need.

## Figures and Tables

**Table 1:** Stage-specific spousal caregiving burden and health impact in dementia.

Dementia Stage	Primary Caregiving Roles	Dominant Burdens	Health Consequences for Spousal Caregiver	Key Intervention Needs
Pre-diagnostic/Early (Mild)	Monitoring subtle cognitive changes; reminders and task support; initiating medical evaluation; emerging surrogate decision-making	Uncertainty; emotional distress; lack of guidance and validation	Anxiety; stress; early depressive symptoms; sleep disturbance; loneliness	Early caregiver education; diagnostic counseling; anticipatory guidance; peer support programs
Middle (Moderate)	Medication and treatment management; assistance with activities of daily living; behavioral symptom management; healthcare system navigation	Escalating time demands; role conflict; loss of intimacy; social withdrawal; physical strain	Depression; anxiety; chronic stress; sleep deprivation; musculoskeletal pain	Skills-based caregiver training; psychosocial interventions; respite services; routine caregiver burden screening
Late (Severe)	24 h supervision; full assistance with personal care; end-of-life and medical decision-making	Anticipatory grief; profound emotional loss; physical exhaustion; financial strain	Frailty and functional decline; immune dysregulation; increased healthcare utilization; possible cognitive decline	Integrated palliative support; formal caregiving assistance; financial and policy support; bereavement counseling
Across All Stages	Continuous emotional and logistical support; advocacy for care recipient	Cumulative caregiver burden; social isolation; stigma	Reduced quality of life; accelerated biological aging; increased morbidity and mortality risk	Stage-sensitive integrated care models; digital health tools; policy reform; caregiver inclusion

Overview of the dementia caregiving trajectory in spousal caregivers, highlighting the evolution of caregiving roles, dominant burden domains, associated health consequences, and corresponding intervention needs across stages of disease progression.

## Data Availability

No new data were created or analyzed in this study. Data sharing is not applicable to this article.
